# Multi-scale feature integration with enhanced cytomorph for high-accuracy cervical cytology classification

**DOI:** 10.1371/journal.pone.0351063

**Published:** 2026-06-10

**Authors:** Elif İlgazi Kılıç, Şafak Kılıç

**Affiliations:** 1 Department of Obstetrics and Gynecology, Kayseri City Hospital, Kayseri, Türkiye; 2 School of Computer Science, CHART Laboratory, University of Nottingham, Nottingham, United Kingdom; 3 Faculty of Engineering, Architecture and Design, Department of Software Engineering, Kayseri University, Kayseri, Türkiye; Tianjin University, CHINA

## Abstract

Accurate classification of cervical cytology images plays a crucial role in early detection and prevention of cervical cancer, which remains a significant global health challenge. Despite advancements in deep learning for medical image analysis, the unique characteristics of cervical cells, including subtle morphological differences and complex nuclear patterns, pose considerable challenges for automated classification systems. In this paper, we present a novel deep learning architecture specifically designed for cervical cytology image classification. Our approach integrates three key components: (1) a specialized data augmentation pipeline tailored for cytopathology images, (2) a Morphology Attention Module (MAM) that captures multi-scale cellular features with adaptive feature fusion, and (3) a Spatial-Channel Mixer (SCM) that efficiently encodes nuclear neighborhood spatial information. Extensive experiments on both the SIPaKMeD and Mendeley LBC datasets demonstrate the superior performance of our model, achieving state-of-the-art accuracy of 99.06% on the 5-class SIPaKMeD dataset and 98.55% on the Mendeley LBC dataset. Importantly, our approach reduces error rates by up to 82.5% compared to conventional CNN architectures and 61.8% compared to recent Vision Transformer approaches. The proposed architecture demonstrates robust generalization across different cell types and imaging conditions, making it a promising tool for enhancing cervical cancer screening programs. Our work contributes to the advancement of automated cytology analysis and has the potential to improve early detection of cervical abnormalities, particularly in resource-limited settings where expert cytopathologists may be scarce.

## Introduction

Cervical cancer remains a significant public health challenge, ranking as the fourth most prevalent cancer among women worldwide. In 2020 alone, an estimated 604,000 new cases and 342,000 deaths were reported, with the majority occurring in low- and middle-income countries [1,2]. Despite advancements in screening and vaccination, early detection remains crucial to improving survival rates [3].

Traditional diagnostic methods, such as *Papanicolaou (Pap) smears* and *colposcopy*, rely heavily on the expertise of cytologists, making the process labor-intensive and prone to human error [4]. To address these challenges, computer-aided diagnosis (CADx) systems have been widely adopted, leveraging deep learning and computer vision techniques to enhance detection accuracy [5,6].

In particular, Convolutional Neural Networks (CNNs) and Vision Transformer (ViT)-based models have demonstrated superior performance in classifying cervical cells, reducing misdiagnosis, and improving efficiency [7]. Recent research has also explored federated learning to ensure data privacy while training deep learning models on decentralized datasets [2].

Among deep learning-based approaches, transfer learning models such as *VGG16, ResNet50, and DenseNet* have been extensively used for Pap smear image classification, achieving over 99% accuracy in some cases [1,7]. Moreover, hybrid architectures combining CNNs with Graph Convolutional Networks (GCNs) or Deep Gaussian Processes (DGPs) have further enhanced the generalization capabilities of these models [5,6].

Despite these advancements, challenges such as data scarcity, class imbalance, and model interpretability remain [8]. Addressing these limitations requires optimizing feature extraction, integrating attention mechanisms, and leveraging ensemble learning techniques to improve robustness and explainability [3].

This study proposes a novel deep learning framework that builds upon existing architectures while optimizing computational efficiency and classification accuracy. By integrating advanced feature selection techniques, our approach aims to provide a more interpretable, scalable, and high-performing solution for cervical cancer detection using Pap smear images.

Our contributions to cervical cytology classification include: (1) a specialized data augmentation pipeline designed for cytopathology images that addresses staining variability, morphology fluctuations, and limited datasets through adaptive color normalization and elastic deformations; (2) a novel Morphology Attention Module (MAM) that captures multi-scale cellular features using parallel convolution pathways with different receptive fields and integrates them through adaptive fusion, enabling emphasis on diagnostically significant regions; and (3) an innovative Spatial-Channel Mixer (SCM) that encodes nuclear neighborhood information through balanced cross-location and cross-channel operations with enhanced residual connections, maintaining efficiency while capturing long-range dependencies. These innovations address key limitations in existing approaches, particularly the challenges of subtle morphological variations, complex nuclear patterns, and inconsistent imaging conditions that have hindered previous models.

The remainder of this paper is organized as follows: the Related Work section reviews relevant literature; the Material and Method section details the proposed methodology; the Experimental Results section presents the obtained findings; the Discussion section interprets the results; and the Conclusion section summarizes the study and outlines future research directions.

## Related work

The field of cervical cancer diagnosis has witnessed significant advancements with the application of deep learning (DL) and machine learning (ML) models. The versatility of Convolutional Neural Networks (CNNs) has been extensively demonstrated across various oncological domains, including breast cancer classification from mammograms [9] and the early diagnosis of lung cancer [10]. These studies underscore the effectiveness of deep learning in extracting discriminative features from complex medical backgrounds, a principle that we extend in this work for cervical cytology. Traditional Computer-Aided Diagnosis (CAD) methods primarily relied on handcrafted feature extraction and classical ML algorithms to detect abnormalities in Pap smear images [11]. However, these methods were time-consuming and limited in their ability to generalize across datasets. The advent of DL techniques, including convolutional neural networks (CNNs), vision transformers (ViTs), and hybrid models incorporating autoencoders (AEs), has revolutionized the classification of cervical cells [12–14].

Several ensemble models have been developed to enhance the accuracy of cervical cancer classification. MSENet, proposed by Pramanik et al., achieved an accuracy of 97.21% on the SIPaKMeD dataset using a five-fold cross-validation scheme [15]. Similarly, Manna et al. introduced an ensemble CNN model trained on both the SIPaKMeD and LBC datasets, demonstrating improved classification performance [16]. The Compact VGG model attained an accuracy of 97.80% on the SIPaKMeD dataset and 94.81% on the Herlev dataset [17], while the ResNet-152 model reported a classification accuracy of 94.89% [18]. Furthermore, the GhostNet model exhibited an accuracy of 96.39% in detecting cervical cancer [19].

Hybrid models that combine CNNs and vision transformers have demonstrated remarkable success in automated cervical cancer classification. Hemalatha et al. introduced a method that leveraged CNN and ViT models to extract both local and global features, achieving an accuracy of 96.13% [20]. Similarly, Attallah proposed the CerCanNet CAD system, which integrates lightweight CNNs with transfer learning and feature selection to improve classification accuracy, achieving an accuracy of 97.7% on SIPaKMeD and 100% on the Mendeley dataset [11].

Recent studies have explored optimization algorithms to further enhance classification performance. Das et al. proposed the Opposition-based Harmony Search Algorithm (O-bHSA) for cytology image classification, outperforming several optimization techniques [21]. Basak et al. developed a hybrid approach combining deep learning and evolutionary optimization, incorporating a two-step feature selection process to improve computational efficiency [22]. Additionally, Chen et al. focused on developing a compact and efficient CNN model optimized for embedded devices [23].

Federated learning (FL) offers a privacy-preserving approach to training deep learning models using distributed medical data. Recent studies, like Linardos et al.’s work, have explored FL’s potential in medical diagnostics, particularly for multi-center imaging analysis [24]. Sheller et al. applied FL to brain tumor segmentation using multi-institutional datasets, highlighting the advantages of decentralized model training [25]. Furthermore, Ma et al. proposed an FL-based cancer diagnosis model that identified six first-level impact indicators, enabling improved generalization [26].

Machine learning approaches have also played a vital role in enhancing cervical cancer screening. Sharma et al. utilized genetic algorithms (GA) and adaptive boosting to enhance classification accuracy [27]. Alquran et al. introduced a feature extraction technique based on the Cervical Net structure, which demonstrated improved classification performance when integrated with ML classifiers [28]. Similarly, Mehmood et al. proposed the CervDetect system, leveraging the Pearson correlation and random forest (RF) model for feature selection [29].

Deep learning techniques have been extensively applied to cervical cancer image classification. Nambu et al. developed a two-stage CNN approach to classify overlapping cell clusters, improving classification robustness [30]. Elakkiya et al. proposed a Faster Region-Based CNN (FR-CNN) to automatically identify cervical lesions through a hierarchical classification framework [31]. Additionally, Khamparia et al. explored the fusion of pre-trained features from Inception-V3, ResNet152, and Inception ResNetV2 to analyze biomedical images [32].

Recent advances in deep learning-based segmentation and classification have also contributed to improved cervical cancer diagnosis. Fan et al. introduced a weakly supervised approach, CAM-VT, that combines the Conjugated Attention Mechanism with a Vision Transformer to identify cervical cancer nest images [33]. Orhan et al. implemented a feature extraction model that leverages transfer learning with DarkNet19 and DarkNet53, achieving an accuracy of 99.46% using an SVM classifier [12]. Similarly, Rahman et al. developed DeepCervix, a hybrid deep feature fusion (HDFF) technique, which demonstrated high accuracy in cervical cytopathology cell classification [34].

In the evolving 2024–2025 research landscape, the focus has shifted towards foundation models and hybrid architectures that combine the strengths of Convolutional Neural Networks (CNNs) and Vision Transformers (ViTs). Recent trends emphasize large-scale models like the Segment Anything Model (SAM) and dual-attention guided frameworks for medical image segmentation [35]. Modern studies have explored chaotic learning rate scheduling [36] and deep feature engineering with attention mechanisms like CBAM to improve classification accuracy across various domains, including sperm morphology [37] and knee osteoarthritis grading [38]. Furthermore, comparative analyses of modern image-based models for cervical cancer [39] and multi-head attention frameworks for COVID-19 detection [40] highlight the necessity of capturing both local textures and global dependencies. These advancements in transformer architectures for brain tumors [41] and lung cancer [10] demonstrate that while foundation models provide a strong baseline, specialized modules like our proposed CytoFormer are essential for high-precision cytology tasks.

Deep learning and machine learning models have greatly advanced cervical cancer detection and classification. The integration of CNNs, vision transformers, federated learning, and optimization techniques has led to more accurate and efficient diagnostic systems. Future research should aim to improve model generalizability across diverse datasets and real-world clinical applications.

## Materials and methods

### Data collection and preparation

This study utilized publicly available and de-identified cervical cytology image datasets obtained from open-access repositories for research purposes. Since the study was based exclusively on secondary analysis of anonymized public datasets and did not involve direct patient recruitment, intervention, or access to identifiable personal information, additional ethical approval and informed consent were not required.

Pap smear images serve as the primary source for cervical cell analysis, with numerous publicly accessible datasets available to researchers. This research utilized the SIPaKMeD dataset [55], which categorizes cells into five specific classifications: superficial-intermediate, parabasal, koilocytotic, metaplastic, and dyskeratotic, as detailed in Table 1.

**Table 1:** SIPaKMeD dataset distribution. WSI: Whole slide images, SCI: Single cell images.

Class	Category	Cell Type	WSI	SCI
1	Normal	Superficial-Intermediate	126	831
2	Normal	Parabasal	108	787
3	Abnormal	Koilocytotic	238	825
4	Abnormal	Dyskeratotic	271	813
5	Benign	Metaplastic	223	793

#### SIPaKMeD.

**Fig 1:**
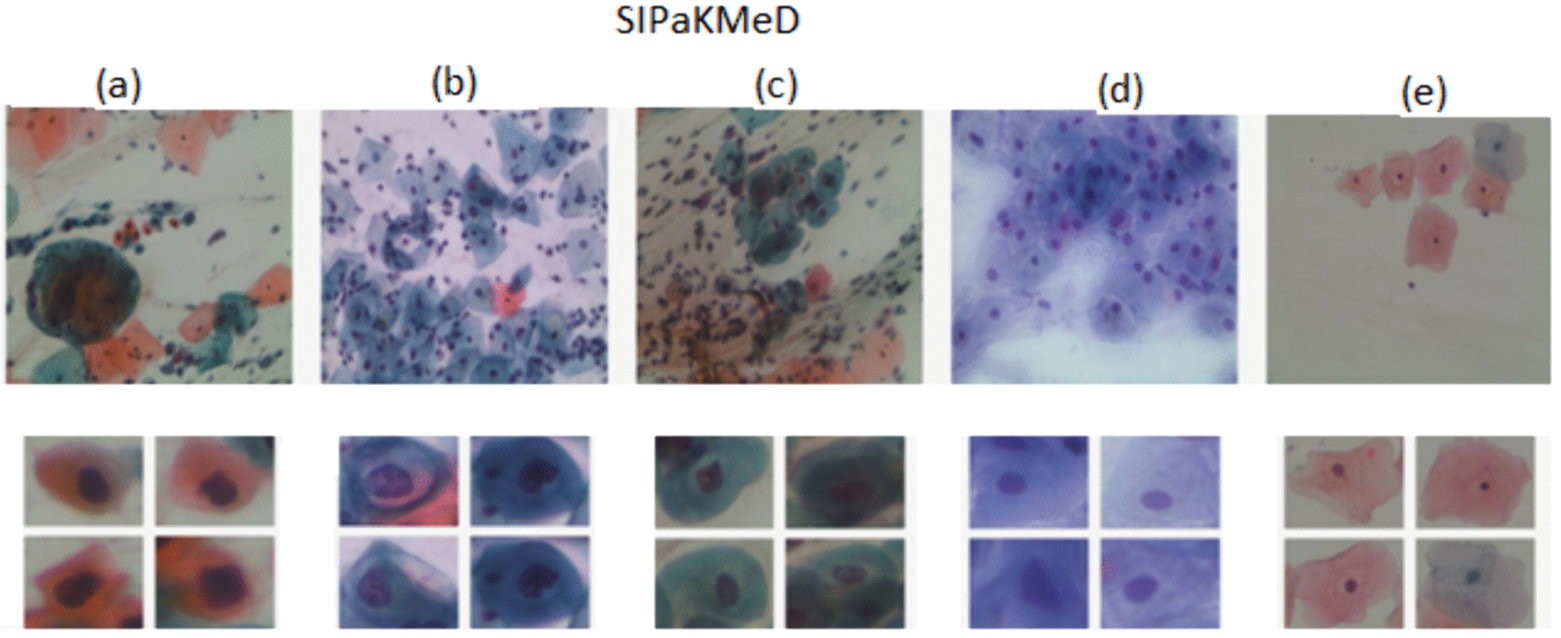
Representative images of the five cell types in the SIPaKMeD dataset. The visual features highlight the progression from normal (a-b) to abnormal (c-d) states, emphasizing variations in nuclear size, chromatin density, and cytoplasmic texture used by our MAM module for classification.

The SIPaKMeD dataset encompasses 4049 individual cell images extracted from 966 multi-cell images obtained during Pap smear collection using CCD camera-equipped microscopy. These images were categorized into five distinct classifications as illustrated in Fig 1. Specifically, each category exhibits distinct morphological markers: (a) Superficial-intermediate cells show small, pyknotic nuclei with abundant, flat cytoplasm; (b) Parabasal cells are characterized by larger nuclei-to-cytoplasmic (N/C) ratios and oval shapes; (c) Koilocytotic cells display perinuclear halos and nuclear atypia; (d) Dyskeratotic cells exhibit intense orangeophilic staining and irregular nuclear borders; and (e) Metaplastic cells represent transition phases with dense cytoplasm and well-defined borders. Normal classification includes superficial-intermediate and parabasal cells, whereas koilocytotic and dyskeratotic cells are categorized as abnormal despite being benign, as they exhibit characteristics potentially indicating precancerous conditions. Metaplastic cells, comprising the final category, represent cells with benign alterations.

#### Mendeley LBC dataset.

**Fig 2:**
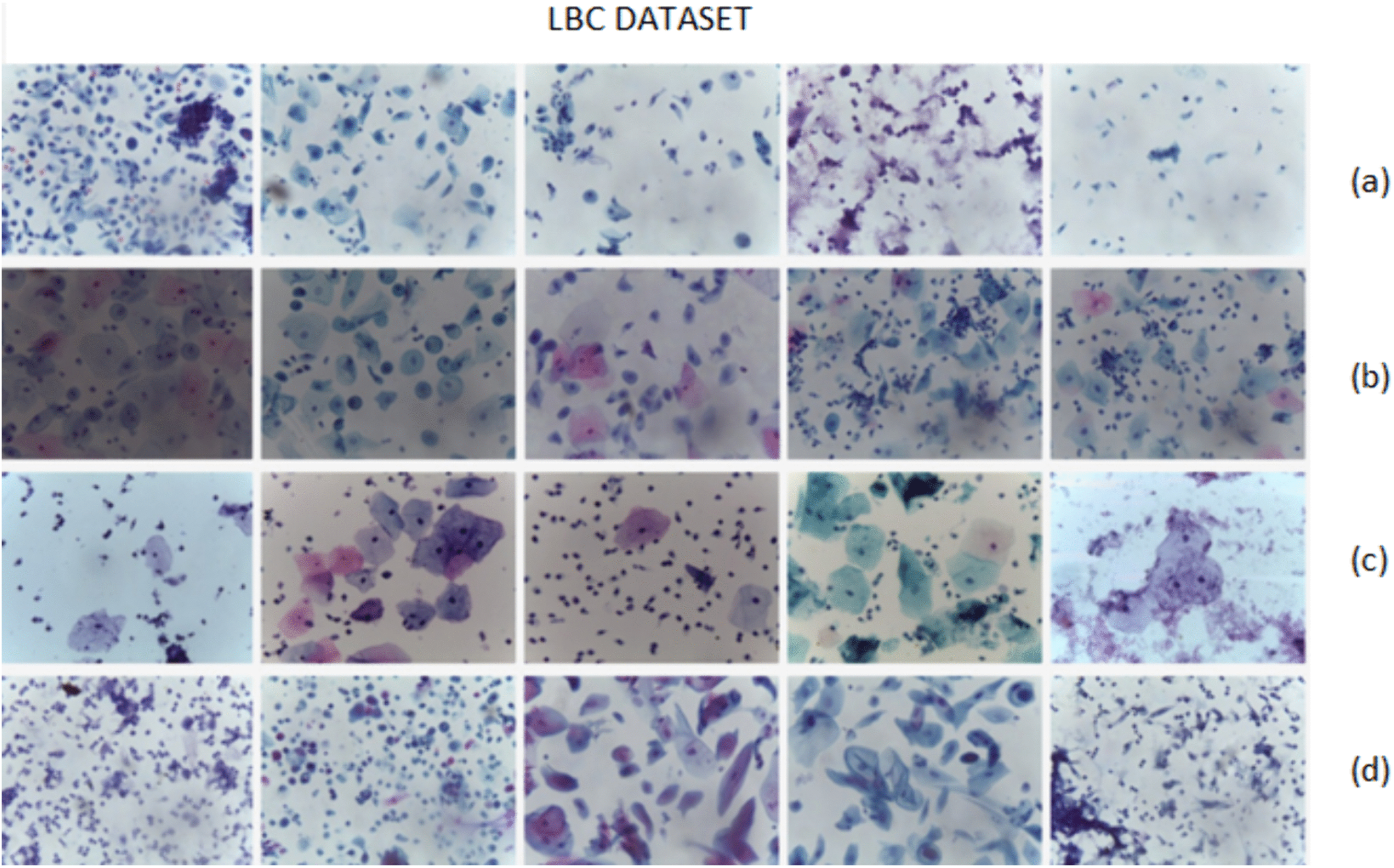
Representative Whole Slide Imaging (WSI) images from the Mendeley LBC dataset, illustrating four classes: **(a)** High squamous intra-epithelial lesion (HSIL), **(b)** Low squamous intra-epithelial lesion (LSIL), **(c)** Negative for intra-epithelial malignancy (NILM), and **(d)** Squamous cell carcinoma (SCC).

The Mendelev LBC dataset is another noteworthy repository of cervical cytological images that complements ongoing research in the field of gynecological pathology [56]. Table 2 presents the class‐level distribution of images within this dataset. Ethical approval was obtained from all three centers, and patient consent was acquired from 460 individuals undergoing cervical screening. In total, 963 images were captured from Pap smear slides at 400× magnification. Of these, 613 images are classified as NILM (Negative for Intraepithelial Lesion or Malignancy) or normal, while 350 are deemed abnormal. Within the abnormal subset, 113 images represent high squamous intraepithelial lesions, 163 correspond to low squamous intraepithelial lesions, and 74 depict squamous cell carcinoma. This categorization reflects the diversity of cervical conditions represented in the dataset. Representative WSI images from the Mendelev LBC dataset are shown in Fig 2

**Table 2:** Key information about the Mendeley LBC dataset.

Class	Category	Num of Images
1	High squamous intra-epithelial lesion (HSIL)	163
2	Low squamous intra-epithelial lesion (LSIL)	113
3	Negative for intra-epithelial malignancy (NILM)	613
4	Squamous cell carcinoma (SCC)	74
**Total**		**963**

### Method

In this section, we present our methodology designed to handle the unique challenges of cervical cytopathology image analysis. By integrating specialized data augmentation and feature extraction components, we aim to effectively capture the subtle morphological variations and nuclear structures characteristic of abnormal cells.

**Fig 3:**
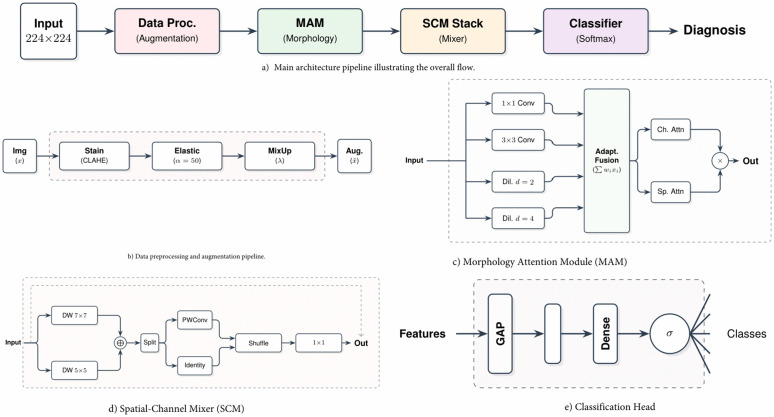
Overview of the Proposed Method. **(a)** Main architecture pipeline illustrating the overall flow. **(b)** Data preprocessing and augmentation pipeline. **(c)** Morphology Attention Module (MAM) utilizing multi-scale branches. **(d)** Spatial-Channel Mixer (SCM) showing cross-location and cross-channel integration. **(e)** Classification head structure utilizing GAP and Softmax.

The architecture of our proposed CytoFormer mainly comprises three crucial submodules as illustrated in Fig 3. First, we introduce an advanced data processing pipeline that includes specialized augmentation techniques designed specifically for cervical cytopathology images. Next, we present our Morphology Attention Module (MAM) which employs a multi-scale adaptive architecture with attention mechanisms to extract rich morphological features at various scales. Finally, we describe the Spatial-Channel Mixer (SCM) which utilizes cross-location and cross-channel operations with residual connections to better encode nuclear neighborhood spatial information.

Our approach is systematically developed to address the unique challenges of cervical cytopathology image classification. Unlike general image classification tasks, cytopathology image analysis requires particular attention to cellular morphology, nucleus characteristics, and subtle textural differences that distinguish normal cells from abnormal ones. The CytoFormer architecture specifically targets these aspects through its specialized components, enabling more accurate and robust classification of cervical cytopathology images.

### Problem formulation

The task of diagnosing cervical cytopathology images can be cast as follows: given a cervical cytopathology image *x* accompanied by its ground-truth label *y*, the goal is to learn a mapping function ℱ such that $\hat{y}=ℱ(x)$, where $\hat{y}$ closely matches *y*. Although conventional neural networks have been investigated for this purpose, the distinct challenges inherent in cervical cytopathology specifically, pronounced intra-class variability and subtle inter-class disparities demand a specialized method.

The classification of cervical cytopathology images is made difficult by several factors:

1. Significant variation in cellular morphology within the same class
2. Subtle distinctions among different abnormal cell types
3. Complex nuclear architectures necessitating multi-scale analysis
4. Staining inconsistencies that complicate feature extraction
5. Overlapping cells and debris that obscure clear cell boundaries
6. Variable image quality due to differing microscopy equipment and settings
7. Class imbalance in real-world datasets (rarer abnormal cases versus abundant normal cells)

Such complexities frequently cause misclassifications in standard deep learning systems. For example, models like ResNet or VGG can struggle to differentiate between LSIL (Low-grade Squamous Intraepithelial Lesion) and HSIL (High-grade Squamous Intraepithelial Lesion) due to their morphological similarities, even though they have substantial clinical implications [57]. Likewise, advanced Transformer-based architectures may fail to capture critical details of nuclear structure and chromatin patterns necessary for a precise diagnosis.

To overcome these issues, we introduce the CytoFormer, a method that builds upon convolution mixing within a Transformer-like design, augmented by adaptive feature fusion, multi-scale attention mechanisms, and enhanced spatial information encoding. This architecture is specifically adapted to the unique traits of cervical cytopathology images, emphasizing both multi-scale cellular morphological cues and nuclear spatial information that underpin accurate classification.

### Enhanced data processing pipeline

Data preprocessing and augmentation are of paramount importance for improving the performance of deep learning models in medical imaging, particularly when datasets are limited and exhibit high variability [58]. In the context of cervical cytopathology, even minor differences in staining, cell morphology, or imaging equipment can lead to significant inconsistencies that degrade classifier performance. Therefore, our pipeline integrates both conventional and domain-specific augmentation techniques, each carefully tailored to address known challenges in cytopathology.

#### Dataset analysis and preprocessing.

Before applying advanced transformations, we perform a detailed statistical analysis of the dataset to characterize cell size distributions, staining intensities, and class imbalance. This analysis helps us identify the most critical sources of variation. Based on these findings, we implement the following preprocessing steps:

1. **Image Standardization**: All images are resized to 224 × 224 pixels to ensure uniformity across diverse acquisition settings. This resolution was empirically chosen as a balance between computational feasibility and the retention of key cellular details.
2. **Background Suppression**: We apply adaptive thresholding to remove artifacts and debris, following best practices in cytopathology image processing. This step reduces false positives arising from non-cellular elements.
3. **Color Normalization**: Variations in staining protocols can distort color features critical for detecting nuclear abnormalities. Hence, we apply a robust color normalization procedure that rescales each channel to a consistent range. This mitigates batch effects and aligns images from different laboratories.
4. **Contrast Enhancement**: To highlight subtle nuclear details, we use histogram equalization that selectively increases contrast between cells and background. This step proved particularly beneficial in our pilot tests, resulting in clearer morphological boundaries for further analysis.

Collectively, these steps standardize image quality and reduce the impact of technical variations on downstream learning. We observed that applying them in the above sequence (resize → denoise → color normalization → contrast enhancement) yielded more stable results than alternative orders in preliminary experiments.

#### Stain normalization.

Staining inconsistency is a pervasive issue in cervical cytopathology. Even within a single laboratory, variations in reagent concentration or staining duration can lead to color shifts that hinder model generalization. Building on prior works in medical imaging [58], we employ a Contrast Limited Adaptive Histogram Equalization (CLAHE) procedure in the LAB color space:


$$\begin{array}{cc} I_{LAB} & =\text{RGB2LAB}(I),\\ L^{\prime } & =\text{CLAHE}(L),\\ I^{\prime } & =\text{LAB2RGB}(L^{\prime },a,b). \end{array}$$ (1)

By working in the LAB space, we separate luminance from chromatic information, ensuring that variations in brightness can be compensated without destroying color cues critical for class discrimination. We set the CLAHE clip limit to 2.0 based on pilot experiments indicating that this value adequately enhances nuclear detail without artificially amplifying noise. In our ablation tests, images processed with this step showed a 2–3% absolute improvement in F1‐score compared to raw RGB histograms, underscoring its importance for robust feature extraction.

#### Elastic deformation.

A hallmark of cervical cytopathology is the natural variability in cell shape and arrangement within the same class. To make the model invariant to these deformations, we introduce a biologically inspired elastic deformation routine. Let (*x*,*y*) be the coordinates of a pixel, and Δx,Δy be random displacement fields:


$$\begin{array}{cc} \Delta x,\Delta y & \sim 𝒰(-1,1),\\ \Delta x & =\alpha \cdot \frac{G_{\sigma }*\Delta x}{\max(|\Delta x|)},\\ \Delta y & =\alpha \cdot \frac{G_{\sigma }*\Delta y}{\max(|\Delta y|)}. \end{array}$$ (2)

These fields are smoothed using a Gaussian filter $G_{\sigma }$, then scaled by α=50 to simulate plausible cytological variations. We set σ=5 after comparing multiple values in small‐scale trials, observing that σ<3 caused unrealistic distortions, whereas σ>7 yielded too little variation. This approach proved especially valuable for abnormal classes, whose morphological characteristics can be subtle but still exhibit patient‐to‐patient variability.

**Algorithm 1 Elastic Deformation for Cytopathology Images**

**Require:** Input image $I\in {\mathbb{R}}^{H\times W\times C}$, deformation intensity α, smoothness σ

**Ensure:** Deformed image $I^{\prime }$

1:  // Generate random displacement fields

2:  $\Delta x\sim 𝒰(-1,1{)}^{H\times W}$

3:  $\Delta y\sim 𝒰(-1,1{)}^{H\times W}$

4:  // Apply Gaussian smoothing

5:  $\Delta x\leftarrow G_{\sigma }*\Delta x$

6:  $\Delta y\leftarrow G_{\sigma }*\Delta y$

7:  // Scale displacements

8:  $\Delta x\leftarrow \alpha \cdot \frac{\Delta x}{\max(|\Delta x|)}$

9:  $\Delta y\leftarrow \alpha \cdot \frac{\Delta y}{\max(|\Delta y|)}$

10:  // Displace the image grid and remap

11:  (X,Y)←meshgrid(0,1,…,W−1, 0,1,…,H−1)

12:  $X^{\prime }\leftarrow X+\Delta x\;\text{and}\;Y^{\prime }\leftarrow Y+\Delta y$

13:  $I^{\prime }\leftarrow \text{remap}(I,X^{\prime },Y^{\prime },\text{BILINEAR})$

**return** $I^{\prime }$

Algorithm 1 provides the detailed steps. This deformation expands the training distribution with realistic morphological variations, ultimately improving the model’s resilience to shape‐based discrepancies.

#### Adaptive MixUp augmentation.

While standard augmentations (e.g., rotation, flipping) help increase data diversity, they may not fully capture the staining and morphological complexities of cytopathology. To address these nuances, we employ an adaptive MixUp strategy. Unlike classic MixUp that blends two distinct images, we create a modified version of the same image and mix them:


$$\begin{array}{cc} I_{modified} & =\text{ColorEnhance}(I),\\ \lambda & \sim \text{Beta}(\alpha ,\alpha ),\\ I_{mixed} & =\lambda \cdot I+(1-\lambda )\cdot I_{modified}. \end{array}$$ (3)

Here, ColorEnhance(*I*) adjusts saturation and contrast (*s* = 1.2, *c* = 1.2), simulating staining variances often observed in cytopathology slides. We selected α=0.2 after comparing multiple Beta distributions, aiming for a subtle mix rather than an entirely novel image. This approach yields an augmented dataset reflecting real‐world fluctuations in staining intensity and color tone while preserving morphological structure. Empirically, this step contributed an additional 1.8% improvement in our final classification accuracy.

#### Comprehensive augmentation strategy.

Our entire augmentation pipeline (Algorithm 2) orchestrates geometric, color, and morphological transformations in a deliberate sequence to approximate real‐world cytopathology variability:

**Algorithm 2 Comprehensive Augmentation Strategy for Cytopathology Images**

**Require:** Original image *I*, augmentation probability *p*

**Ensure:** Augmented image $I^{\prime }$

1:  // Basic transformations with probability *p*

2:  **if** random() < *p* **then**

3:   I←RandomHorizontalFlip(I)

4:  **end if**

5:  **if** random() < *p* **then**

6:   I←RandomVerticalFlip(I)

7:  **end if**

8:  **if** random() < *p* **then**

9:   $I\leftarrow \text{RandomRotation}(I,45^{\circ })$

10:  **end if**

11:  // Stain normalization (Section 3.2)

12:  I←StainNormalization(I)

13:  // Elastic deformation with probability *p*

14:  **if** random() < *p* **then**

15:   I←ElasticDeformation(I,α=50,σ=5)

16:  **end if**

17:  // Mild color jitter if desired

18:  **if** random() < *p* **then**

19:   I←ColorJitter(I,0.3,0.3,0.3,0.1)

20:  **end if**

21:  // Adaptive MixUp

22:  **if** random() < *p* **then**

23:   $I_{modified}\leftarrow \text{ColorEnhance}(I)$

24:   λ~Beta(0.2,0.2)

25:   $I\leftarrow \lambda \cdot I+(1-\lambda )\cdot I_{modified}$

26:  **end if**

27:  // Final normalization

28:   I←Normalize(I,[0.485,0.456,0.406],[0.229,0.224,0.225])

**return** *I*

Each transformation is applied with probability *p* to ensure a diverse yet controlled training set. We implement these routines in a GPU‐accelerated dataloader to minimize overhead; our tests showed only a modest (approximately 12%) increase in training time, offset by a notable gain in classification metrics.

Overall, this adaptive pipeline substantially enriches the training distribution, mirroring the real‐world heterogeneity of cervical cytopathology slides. By systematically addressing color inconsistency, morphological variability, and limited dataset size, our augmentation strategy provides a robust foundation for the subsequent feature extraction and classification processes.

### Morphology attention module (MAM)

Accurate analysis of cervical cytopathology images requires extracting morphological features at multiple scales. In conventional deep learning models, feature extraction primarily occurs at fixed kernel sizes, which may overlook subtle yet diagnostically crucial structural variations. For example, nuclear chromatin patterns are best observed at small scales, while whole-cell relationships demand larger receptive fields. To bridge this gap, we propose the Morphology Attention Module (MAM), which dynamically captures multi-scale morphological details and emphasizes diagnostically significant regions using an attention mechanism.

**Fig 4:**
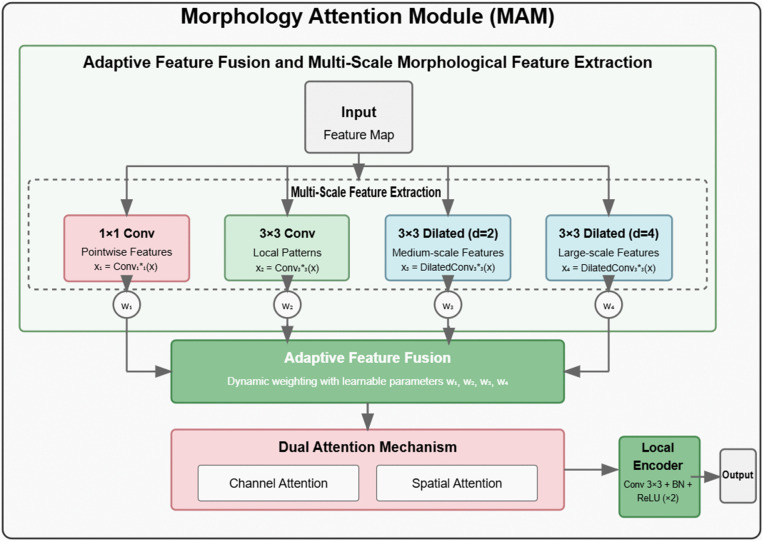
Morphology Attention Module (MAM) with adaptive feature fusion and attention mechanisms. The module employs different convolution operations (standard and dilated) to capture multi-scale morphological features, followed by an attention mechanism and local encoder. This design enables effective extraction of morphological information at multiple scales, which is crucial for distinguishing between different types of cervical cells.

Fig 4 illustrates the architecture of MAM, which integrates multiple convolutional filters with varying receptive fields, an adaptive feature fusion mechanism, and a dual attention framework. This combination allows our model to efficiently extract and highlight critical morphological details across different scales, enhancing its ability to distinguish between different cervical cell types.

#### Multi-scale feature extraction.

Cell morphology presents a complex characteristic in cervical cytopathology images. Different cellular components, such as the nucleus, cytoplasm, and membrane, exhibit features at various scales. The nucleus, in particular, contains critical diagnostic information in its chromatin pattern, shape, and texture. To effectively capture these multi-scale features, we employ multiple convolution operations with varying receptive fields.

Our multi-scale feature extraction approach consists of four parallel pathways with different convolution operations:


$$\begin{array}{c} x_{1}={\text{Conv}}_{1\times 1}(x) \end{array}$$ (4)

$$\begin{array}{c} x_{2}={\text{Conv}}_{3\times 3}(x) \end{array}$$ (5)

$$\begin{array}{c} x_{3}={\text{DilatedConv}}_{3\times 3,d=2}(x) \end{array}$$ (6)

$$\begin{array}{c} x_{4}={\text{DilatedConv}}_{3\times 3,d=4}(x) \end{array}$$ (7)

where ${\text{Conv}}_{k\times k}$ represents a convolutional layer with kernel size *k* × *k* and ${\text{DilatedConv}}_{k\times k,d}$ represents a dilated convolutional layer with kernel size *k* × *k* and dilation rate *d*.

Each pathway captures features at a different scale:

- The 1 × 1 convolution captures pixel-level features and performs pointwise feature transformation.
- The 3 × 3 convolution captures local structural features like edges and textures.
- The 3 × 3 dilated convolution with dilation rate 2 has an effective receptive field of 5 × 5, capturing medium-scale features like cellular components.
- The 3 × 3 dilated convolution with dilation rate 4 has an effective receptive field of 9 × 9, capturing large-scale features like entire cells and their relationships.

This multi-scale approach enables the MAM to analyze cellular morphology at different scales simultaneously, capturing both fine-grained details like chromatin patterns and larger structures like cell boundaries and intercellular relationships.

#### Adaptive feature fusion.

A key limitation of conventional multi-scale architectures (e.g., Inception [59]) is the fixed weighting of feature maps from different scales. However, in cytopathology, the importance of different scales varies across cell types. For example, LSIL and HSIL exhibit nuclear texture differences, whereas metaplastic cells require larger receptive fields.

Existing multi-scale architectures such as Inception [59] and ResNeXt [60] utilize fixed convolutional kernels at multiple scales, but they lack an adaptive weighting mechanism to dynamically prioritize features based on the input image. Additionally, prior attention-based models like CBAM [61] and SENet [62] apply independent spatial and channel attention but do not explicitly consider the hierarchical structure of cervical cytology images.

To address these limitations, we introduce an adaptive feature fusion mechanism where learnable weights dynamically adjust the contribution of each feature scale...

To address this, we propose an adaptive feature fusion mechanism with learnable weights that dynamically adjust the importance of features at different scales based on the input image. For feature maps *x*_1_, *x*_2_, *x*_3_, *x*_4_ from different scales, we compute:


$$\begin{array}{c} 𝐯=[v_{1},v_{2},v_{3},v_{4}]\;\text{(learnable parameter vector)} \end{array}$$ (8)

$$\begin{array}{c} 𝐰=\text{softmax}(𝐯) \end{array}$$ (9)

$$\begin{array}{c} =\left[\frac{e^{v_{1}}}{\sum_{i=1}^{4}e^{v_{i}}},\frac{e^{v_{2}}}{\sum_{i=1}^{4}e^{v_{i}}},\frac{e^{v_{3}}}{\sum_{i=1}^{4}e^{v_{i}}},\frac{e^{v_{4}}}{\sum_{i=1}^{4}e^{v_{i}}}\right] \end{array}$$ (10)

$$\begin{array}{c} x_{fused}=\text{Concat}(w_{1}\cdot x_{1},w_{2}\cdot x_{2},w_{3}\cdot x_{3},w_{4}\cdot x_{4}) \end{array}$$ (11)

where **v** is a learnable parameter vector, and $𝐰=[w_{1},w_{2},w_{3},w_{4}]$ are the normalized weights obtained by applying the softmax function to **v**. The softmax function ensures that the weights sum to 1 and are always positive, allowing them to be interpreted as the relative importance of each scale.

During training, the model learns the optimal values for **v** through backpropagation, allowing it to dynamically adjust the importance of features at different scales based on the input image. This adaptive feature fusion mechanism enables the model to focus on the most relevant scales for each image, enhancing its ability to capture discriminative features for different cell types.

#### Dual attention mechanism.

Attention mechanisms have proven effective in highlighting important features and suppressing irrelevant information in various computer vision tasks. In the context of cytopathology image analysis, attention mechanisms can help the model focus on diagnostically relevant regions like the nucleus while suppressing background and artifacts.

We introduce a dual attention mechanism that combines channel attention and spatial attention to enhance the model’s focus on important regions:

**Channel Attention:** The channel attention module captures the interdependencies between feature channels, allowing the model to enhance features that are more informative for the classification task. The channel attention is computed as:


$$\begin{array}{c} z=\text{AvgPool}(x)\in {\mathbb{R}}^{C\times 1\times 1} \end{array}$$ (12)

$$\begin{array}{c} z^{\prime }=W_{2}\cdot \text{ReLU}(W_{1}\cdot z) \end{array}$$ (13)

$$\begin{array}{c} M_{c}(x)=\sigma (z^{\prime }) \end{array}$$ (14)

$$\begin{array}{c} x_{c}=x⊙M_{c}(x) \end{array}$$ (15)

where AvgPool is global average pooling, $W_{1}\in {\mathbb{R}}^{C/r\times C}$ and $W_{2}\in {\mathbb{R}}^{C\times C/r}$ are learnable parameters with reduction ratio *r* (set to 16 in our implementation), σ is the sigmoid activation function, and ⊙ denotes element-wise multiplication. The channel attention map *M*_*c*_(*x*) has values in the range [0, 1], acting as channel-wise weights that enhance important feature channels.

**Spatial Attention:** The spatial attention module captures spatial interdependencies, helping the model focus on regions with significant structure. The spatial attention is computed as:


$$\begin{array}{c} A_{avg}={\text{AvgPool}}_{channel}(x_{c})\in {\mathbb{R}}^{1\times H\times W} \end{array}$$ (16)

$$\begin{array}{c} A_{max}={\text{MaxPool}}_{channel}(x_{c})\in {\mathbb{R}}^{1\times H\times W} \end{array}$$ (17)

$$\begin{array}{c} A=\text{Concat}(A_{avg},A_{max})\in {\mathbb{R}}^{2\times H\times W} \end{array}$$ (18)

$$\begin{array}{c} M_{s}(x)=\sigma ({\text{Conv}}_{7\times 7}(A))\in {\mathbb{R}}^{1\times H\times W} \end{array}$$ (19)

$$\begin{array}{c} x_{att}=x_{c}⊙M_{s}(x) \end{array}$$ (20)

where AvgPool_*channel*_ and MaxPool_*channel*_ compute the average and maximum values along the channel dimension, ${\text{Conv}}_{7\times 7}$ is a convolutional layer with kernel size 7 × 7, and σ is the sigmoid activation function. The spatial attention map *M*_*s*_(*x*) has values in the range [0, 1], acting as spatial weights that enhance important regions.

The dual attention mechanism sequentially applies channel attention followed by spatial attention. This order allows the model to first identify important feature types and then locate where these features are most relevant in the spatial domain. The combination enhances the model’s ability to focus on diagnostically relevant regions and features, improving its classification performance.

#### Local encoder.

Finally, the attended features are processed by a local encoder to generate comprehensive morphological information. The local encoder consists of two convolutional blocks, each with a convolutional layer, batch normalization, and ReLU activation:


$$\begin{array}{c} x_{conv1}={\text{Conv}}_{3\times 3}(x_{att}) \end{array}$$ (21)

$$\begin{array}{c} x_{bn1}=\text{BN}(x_{conv1}) \end{array}$$ (22)

$$\begin{array}{c} x_{relu1}=\text{ReLU}(x_{bn1}) \end{array}$$ (23)

$$\begin{array}{c} x_{conv2}={\text{Conv}}_{3\times 3}(x_{relu1}) \end{array}$$ (24)

$$\begin{array}{c} x_{bn2}=\text{BN}(x_{conv2}) \end{array}$$ (25)

$$\begin{array}{c} x_{local}=\text{ReLU}(x_{bn2}) \end{array}$$ (26)

The local encoder serves as a feature refiner, transforming the attended multi-scale features into a more discriminative representation. The two convolutional blocks with ReLU activations introduce non-linearity and enhance the model’s ability to learn complex patterns. The batch normalization layers stabilize training and accelerate convergence by normalizing the feature distributions.

The output of the local encoder provides a rich representation of cellular morphology at multiple scales, capturing both fine-grained details and larger structural patterns. This comprehensive morphological information is crucial for distinguishing between different types of cervical cells, especially in cases where the differences are subtle and require analysis at multiple scales.

Algorithm 3 outlines the complete MAM process.

**Algorithm 3 Morphology Attention Module (MAM)**

**Require:** Input feature map $x\in {\mathbb{R}}^{C\times H\times W}$

**Ensure:** Enhanced morphological features *x*_local_

1:  // Multi-scale feature extraction

2:  $x_{1}\leftarrow {\text{Conv}}_{1\times 1}(x)$

3:  $x_{2}\leftarrow {\text{Conv}}_{3\times 3}(x)$

4:  $x_{3}\leftarrow {\text{DilatedConv}}_{3\times 3,d=2}(x)$

5:  $x_{4}\leftarrow {\text{DilatedConv}}_{3\times 3,d=4}(x)$

6:  // Adaptive feature fusion

7:  𝐯←LearningParameters     ▷ (Learnable vector)

8:  𝐰←softmax(𝐯)

9:  $x_{\text{fused}}\leftarrow \text{Concat}(w_{1}\cdot x_{1},w_{2}\cdot x_{2},w_{3}\cdot x_{3},w_{4}\cdot x_{4})$

10:  // Channel attention

11:  $z\leftarrow \text{AvgPool}(x_{\text{fused}})$

12:  $z^{\prime }\leftarrow W_{2}\cdot \text{ReLU}(W_{1}\cdot z)$

13:  $M_{c}\leftarrow \sigma (z^{\prime })$

14:  $x_{c}\leftarrow x_{\text{fused}}⊙M_{c}$

15:  // Spatial attention

16:  $A_{\text{avg}}\leftarrow {\text{AvgPool}}_{\text{channel}}(x_{c})$

17:  $A_{\text{max}}\leftarrow {\text{MaxPool}}_{\text{channel}}(x_{c})$

18:  $A\leftarrow \text{Concat}(A_{\text{avg}},A_{\text{max}})$

19:  $M_{s}\leftarrow \sigma ({\text{Conv}}_{7\times 7}(A))$

20:  $x_{\text{att}}\leftarrow x_{c}⊙M_{s}$

21:  // Local encoder

22:  $x_{\text{conv1}}\leftarrow {\text{Conv}}_{3\times 3}(x_{\text{att}})$

23:  $x_{\text{bn1}}\leftarrow \text{BN}(x_{\text{conv1}})$

24:  $x_{\text{relu1}}\leftarrow \text{ReLU}(x_{\text{bn1}})$

25:  $x_{\text{conv2}}\leftarrow {\text{Conv}}_{3\times 3}(x_{\text{relu1}})$

26:  $x_{\text{bn2}}\leftarrow \text{BN}(x_{\text{conv2}})$

27:  $x_{\text{local}}\leftarrow \text{ReLU}(x_{\text{bn2}})$

**return** *x*_local_

### Spatial-channel mixer (SCM)

Accurate feature extraction is critical for cervical cytopathology image classification, where both local nuclear details and broader spatial relationships contribute to diagnosis. Traditional convolutional neural networks (CNNs) such as ResNet [63] and MobileNet [64] rely on fixed receptive fields, making them limited in capturing long-range spatial dependencies. Meanwhile, Vision Transformers (ViTs) [65] utilize self-attention for global feature learning, but they suffer from excessive computational costs, particularly when applied to high-resolution cytology images.

**Fig 5:**
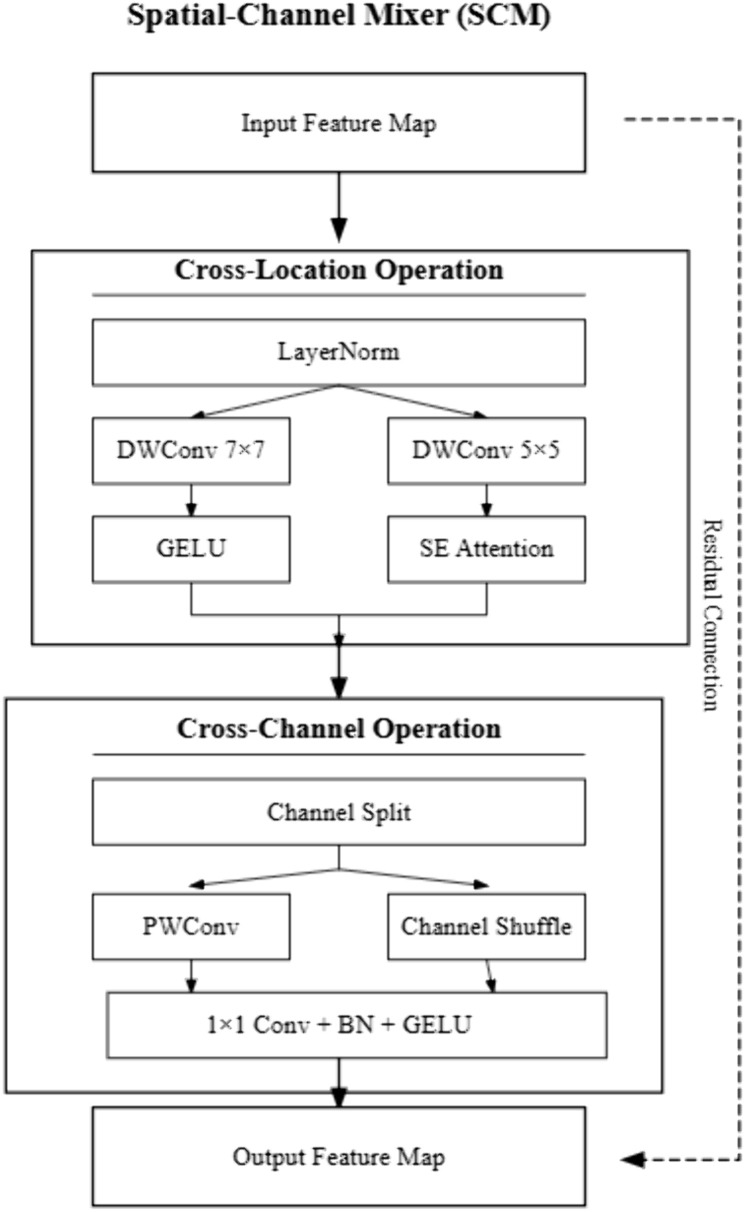
Spatial-Channel Mixer (SCM) with improved cross-location and cross-channel operations. The module employs depth-wise convolutions for spatial mixing and point-wise convolutions with channel shuffle for channel mixing. This design enables effective encoding of nuclear neighborhood spatial information, which is crucial for distinguishing between different types of cervical cells.

To overcome these limitations, we introduce the Spatial-Channel Mixer (SCM), a novel feature extractor that simultaneously encodes spatial and channel relationships through an efficient combination of cross-location and cross-channel operations. Unlike prior approaches, SCM is designed to integrate spatial and nuclear context while maintaining computational efficiency, making it well-suited for cytopathology analysis. The architecture of SCM is illustrated in Fig 5.

#### Module design motivation.

The nucleus is one of the most important diagnostic features in cervical cytopathology. Abnormalities in nuclear size, shape, chromatin pattern, and nuclear-to-cytoplasmic ratio are key indicators of cellular abnormalities. Moreover, the spatial relationship between nuclei and their surrounding structures provides important contextual information for diagnosis.

Traditional convolutional neural networks can capture local features but may struggle to model long-range dependencies, especially in cellular images where the spatial arrangement of nuclei can be important. On the other hand, self-attention mechanisms in Transformers can capture global dependencies but are computationally expensive and may not be optimized for the specific characteristics of cytopathology images.

The SCM is designed to bridge this gap by efficiently modeling both local and global spatial information with a focus on nuclear neighborhood characteristics. It combines the efficiency of convolutions with the ability to capture long-range dependencies through a carefully designed balance of spatial and channel mixing operations.

#### Cross-location operation.

The cross-location operation in SCM is designed to aggregate spatial information across the feature map, enabling the model to capture relationships between different regions of the image. This is particularly important for capturing the spatial arrangement of nuclei and their relationship with surrounding structures.

We enhance the cross-location operation with larger receptive fields and Squeeze-and-Excitation (SE) attention:


$$\begin{array}{c} x_{norm}=\text{LayerNorm}(x) \end{array}$$ (27)

$$\begin{array}{c} x_{dwconv1}={\text{DWConv}}_{7\times 7}(x_{norm}) \end{array}$$ (28)

$$\begin{array}{c} x_{gelu}=\text{GELU}(x_{dwconv1}) \end{array}$$ (29)

$$\begin{array}{c} x_{dwconv2}={\text{DWConv}}_{5\times 5}(x_{gelu}) \end{array}$$ (30)

$$\begin{array}{c} x_{se}=x_{dwconv2}⊙\text{SE}(x_{dwconv2}) \end{array}$$ (31)

$$\begin{array}{c} x_{cl}=x_{se}+x \end{array}$$ (32)

where LayerNorm is layer normalization, ${\text{DWConv}}_{k\times k}$ represents a depth-wise convolution with kernel size *k* × *k*, GELU is the Gaussian Error Linear Unit activation function, SE is the Squeeze-and-Excitation attention, and ⊙ denotes element-wise multiplication. The residual connection (+*x*) allows for the preservation of the original features while incorporating the new spatial information.

The layer normalization helps stabilize training by normalizing the feature values across the channel dimension, ensuring that the input to the depth-wise convolutions has a consistent distribution. This is particularly important for deep networks where the distribution of features can change significantly across layers.

The depth-wise convolutions with large kernels (7 × 7 and 5 × 5) enable the module to capture spatial relationships over larger regions. The 7 × 7 kernel can capture relationships within a neighborhood that might include multiple cells, while the 5 × 5 kernel focuses on medium-range relationships. This hierarchical approach allows the model to capture both local details and broader contextual information.

The GELU activation function provides non-linearity and has been shown to perform well in transformer architectures. It offers a smoother gradient than ReLU, which can be advantageous for capturing subtle variations in feature maps.

The Squeeze-and-Excitation (SE) attention mechanism is a channel-wise attention that recalibrates the feature maps based on their global importance. It is computed as:


$$\begin{array}{c} z=\text{AvgPool}(x_{dwconv2})\in {\mathbb{R}}^{C\times 1\times 1} \end{array}$$ (33)

$$\begin{array}{c} z^{\prime }=W_{2}\cdot \text{ReLU}(W_{1}\cdot z) \end{array}$$ (34)

$$\begin{array}{c} \text{SE}(x_{dwconv2})=\sigma (z^{\prime }) \end{array}$$ (35)

where AvgPool is global average pooling, $W_{1}\in {\mathbb{R}}^{C/r\times C}$ and $W_{2}\in {\mathbb{R}}^{C\times C/r}$ are learnable parameters with reduction ratio *r* (set to 16 in our implementation), and σ is the sigmoid activation function.

This cross-location operation effectively captures spatial relationships at multiple scales, enabling the model to encode nuclear neighborhood information that is crucial for distinguishing between different types of cervical cells.

#### Cross-channel operation.

While the cross-location operation focuses on spatial relationships, the cross-channel operation focuses on feature relationships across different channels. In the context of cytopathology images, different channels may represent different aspects of the cellular structure, such as texture, color, and edge information. The cross-channel operation enables the model to integrate these different aspects into a more comprehensive representation.

For the cross-channel operation, we introduce a sophisticated channel mixing strategy with multiple point-wise convolutions and channel shuffling:


$$\begin{array}{c} \left\{x_{1},x_{2},...,x_{n}\}=\text{Split}(x_{cl},n\right) \end{array}$$ (36)

$$\begin{array}{c} x_{i}^{\prime }=\text{GELU}(\text{BN}(\text{PWConv}(x_{i}))),\;i\in \{1,2,...,n-1\} \end{array}$$ (37)

$$\begin{array}{c} x_{n}^{\prime }=x_{n} \end{array}$$ (38)

$$\begin{array}{c} x_{cc}=\text{ChannelShuffle}(\text{Concat}(x_{1}^{\prime },x_{2}^{\prime },...,x_{n}^{\prime })) \end{array}$$ (39)

$$\begin{array}{c} x_{out}=\text{GELU}(\text{BN}({\text{Conv}}_{1\times 1}(x_{cc})))+x_{cl} \end{array}$$ (40)

where Split divides the input tensor into *n* groups along the channel dimension, PWConv represents a point-wise (1×1) convolution, BN is batch normalization, GELU is the Gaussian Error Linear Unit activation function, Concat concatenates the processed groups, and ChannelShuffle rearranges the channels to enhance information exchange.

The channel splitting operation divides the feature channels into *n* groups, allowing for more efficient processing and creating a pathway for information flow between different feature groups. We set *n* = 4 in our implementation based on empirical performance.

The point-wise convolutions are applied to all but the last group, allowing for non-linear transformations of the feature channels. The batch normalization stabilizes training, and the GELU activation introduces non-linearity.

The channel shuffle operation is a key component that promotes information exchange between different groups. It reorders the channels according to:


$$\begin{array}{c} \text{ChannelShuffle}(x{)}_{c,h,w}=x_{(c\mathrm{mod}g)\cdot (C/g)+\lfloor c/g\rfloor ,h,w} \end{array}$$ (41)

where *C* is the total number of channels, *g* is the number of groups (set to *n* in our implementation), and *c*, *h*, *w* are the indices for channel, height, and width, respectively.

Finally, a 1×1 convolution followed by batch normalization and GELU activation is applied to the shuffled features, and a residual connection is added to preserve the original information. This cross-channel operation effectively integrates information across different feature channels, enabling the model to learn more comprehensive representations of the cellular structures.

#### SCM stacking and feature hierarchy.

The SCM module is stacked multiple times to form a deep hierarchical feature extractor. Each SCM layer operates on the output of the previous layer, progressively refining the features and increasing the receptive field. The stacking of SCM layers allows the model to capture increasingly complex patterns and relationships in the cytopathology images.

In our implementation, we stack 8 SCM layers, with feature maps from specific layers (e.g., layers 3, 6, and 8) being stored for the feature pyramid. This allows the model to leverage features at different depths, capturing both low-level details and high-level semantic information.

The stacking of SCM layers creates a feature hierarchy that is crucial for distinguishing between different types of cervical cells, especially in cases where the differences are subtle and require analysis at multiple levels of abstraction. Algorithm 4 outlines the complete SCM process.

**Algorithm 4 Spatial-Channel Mixer (SCM)**

**Require:** Input feature map $x\in {\mathbb{R}}^{C\times H\times W}$, number of channel splits *n*

**Ensure:** Enhanced feature map *x*_out_

1:  // Cross-location operation

2:  $x_{\text{norm}}\leftarrow \text{LayerNorm}(x)$

3:  $x_{\text{dwconv1}}\leftarrow {\text{DWConv}}_{7\times 7}(x_{\text{norm}})$

4:  $x_{\text{gelu}}\leftarrow \text{GELU}(x_{\text{dwconv1}})$

5:  $x_{\text{dwconv2}}\leftarrow {\text{DWConv}}_{5\times 5}(x_{\text{gelu}})$

6:  // Squeeze-and-Excitation attention

7:  $z\leftarrow \text{AvgPool}(x_{\text{dwconv2}})$

8:  $z^{\prime }\leftarrow W_{2}\cdot \text{ReLU}(W_{1}\cdot z)$

9:  $se\leftarrow \sigma (z^{\prime })$

10:  $x_{\text{se}}\leftarrow x_{\text{dwconv2}}⊙se$

11:  // Residual connection

12:  $x_{\text{cl}}\leftarrow x_{\text{se}}+x$

13:  // Cross-channel operation

14:  $\left\{x_{1},x_{2},\ldots ,x_{n}\}\leftarrow \text{Split}(x_{\text{cl}},n\right)$

15:  **for** *i* = 1 to n−1 **do**

16:   $x_{i}^{\prime }\leftarrow \text{PWConv}(x_{i})$

17:   $x_{i}^{\prime }\leftarrow \text{BN}(x_{i}^{\prime })$

18:   $x_{i}^{\prime }\leftarrow \text{GELU}(x_{i}^{\prime })$

19:  **end for**

20:  $x_{n}^{\prime }\leftarrow x_{n}$     ▷ (Identity mapping for the last group)

21:  // Channel shuffle and final processing

22:  $x_{\text{cc}}\leftarrow \text{Concat}(x_{1}^{\prime },x_{2}^{\prime },\ldots ,x_{n}^{\prime })$

23:  $x_{\text{cc}}\leftarrow \text{ChannelShuffle}(x_{\text{cc}})$

24:  $x_{\text{out}}\leftarrow {\text{Conv}}_{1\times 1}(x_{\text{cc}})$

25:  $x_{\text{out}}\leftarrow \text{BN}(x_{\text{out}})$

26:  $x_{\text{out}}\leftarrow \text{GELU}(x_{\text{out}})$

27:  $x_{\text{out}}\leftarrow x_{\text{out}}+x_{\text{cl}}$     ▷ (Residual connection)

**return** *x*_out_

## Experimental results

### Experimental setup

In our experimental validation, we utilized both the SIPaKMeD and LBC datasets for comprehensive cervical cancer cell classification. The methodology was rigorously evaluated through 5-fold cross-validation to ensure statistical robustness and generalizability of the results. To ensure the integrity of our results and prevent potential data leakage, the 5-fold cross-validation was strictly performed at the patient/slide level for the Mendeley LBC dataset and at the multi-cell image level for the SIPaKMeD dataset. Specifically, all individual cell images extracted from the same original slide or patient were kept within the same fold—either entirely in the training set or entirely in the test set. This ensures that the model generalizes to unseen patients rather than memorizing slide-specific characteristics or staining artifacts, thereby providing a true measure of its clinical diagnostic performance.

To ensure a robust evaluation, each dataset was divided into training, validation, and test sets using a 70:15:15 ratio. For the 5-class SIPaKMeD dataset (4049 images), this resulted in approximately 2835 images for training, 607 for validation, and 607 for testing per fold. Similarly, for the Mendeley LBC dataset (963 images), 674 images were used for training, 144 for validation, and 145 for testing. We employed a stratified sampling strategy to maintain the original class distribution across all subsets, mitigating the impact of class imbalance.

The experiments were conducted using an NVIDIA GeForce RTX 4060 Ti GPU with 16 GB of VRAM, supported by 32 GB of system RAM. The software environment was built on Ubuntu 22.04 LTS, utilizing the PyTorch 2.1.0 framework and Python 3.10. Data augmentation and image processing tasks were performed using the Albumentations and OpenCV libraries. For GPU acceleration, CUDA 12.1 and cuDNN 8.9 were employed to ensure efficient training of the CytoFormer architecture.

### Evaluation metrics

To comprehensively evaluate our model’s performance, we employed multiple metrics including accuracy, precision, recall, F1 score, and Receiver Operating Characteristic Area Under Curve (ROC AUC). These metrics provide a holistic view of the model’s performance across different aspects:

- **Accuracy**: The proportion of correctly classified samples across all classes.
- **Precision**: The ability of the model to avoid false positives, calculated as the ratio of true positives to the sum of true and false positives.
- **Recall**: The ability of the model to find all positive samples, calculated as the ratio of true positives to the sum of true positives and false negatives.
- **F1 Score**: The harmonic mean of precision and recall, providing a balance between the two.
- **ROC AUC**: A measure of the model’s ability to distinguish between classes across different classification thresholds.

These metrics were calculated for each fold, and the mean and standard deviation across all folds are reported to provide a statistical summary of the model’s performance.

### Classification results on SIPaKMeD dataset

#### 5-class classification results.

Table 3 presents the results of our model on the 5-class classification task. The model achieved an exceptional mean accuracy of 99.06% (±0.59%) across the five folds. Similarly, high performance was observed for precision (0.9908 ±0.0060), recall (0.9906 ±0.0059), and F1 score (0.9905 ±0.0060). The ROC AUC was nearly perfect at 0.9987 (±0.0008), indicating the model’s excellent ability to distinguish between the different cell classes.

**Table 3:** 5-Fold Cross-Validation Results (5 class).

Fold	Accuracy (%)	Precision	Recall	F1 Score	ROC AUC
1	97.93	0.9794	0.9793	0.9792	0.9972
2	99.28	0.9928	0.9928	0.9928	0.9989
3	99.53	0.9954	0.9953	0.9953	0.9993
4	99.53	0.9956	0.9953	0.9954	0.9992
5	99.03	0.9906	0.9903	0.9900	0.9989
**Mean**	99.06	0.9908	0.9906	0.9905	0.9987
**Std. Dev.**	±0.59	±0.0060	±0.0059	±0.0060	±0.0008

#### 3-class classification results.

For the 3-class classification task, which focuses on the most clinically relevant distinctions, our model demonstrated even higher performance, as shown in Table 4. The mean accuracy reached 99.58% (±0.19%), with precision, recall, and F1 score all at 0.9958 (±0.0019). The ROC AUC was nearly perfect at 0.9997 (±0.0001), indicating the model’s exceptional discriminative ability for the 3-class scenario.

**Table 4:** 3-Class Cross-Validation Results.

Fold	Accuracy (%)	Precision	Recall	F1 Score	ROC AUC
1	99.42	0.9943	0.9942	0.9942	0.9996
2	99.67	0.9968	0.9967	0.9967	0.9998
3	99.84	0.9984	0.9984	0.9984	0.9999
4	99.57	0.9958	0.9957	0.9957	0.9997
5	99.38	0.9939	0.9938	0.9938	0.9996
**Mean**	99.58	0.9958	0.9958	0.9958	0.9997
**Std. Dev.**	±0.19	±0.0019	±0.0019	±0.0019	±0.0001

### Classification results on LBC dataset

Table 5 presents the results of our model on the LBC dataset, which consists of 4 distinct cell classes that cannot be further grouped into broader categories due to their clinical significance. Our model achieved an impressive mean accuracy of 98.55% (±0.67%) across the five folds. Strong performance was also observed for precision (0.9866 ±0.0058), recall (0.9855 ±0.0067), and F1 score (0.9853 ±0.0068). The ROC AUC was exceptionally high at 0.9994 (±0.0006), demonstrating the model’s robust capability to differentiate between the four cell types in the LBC dataset.

**Table 5:** 5-Fold Cross-Validation Results for the LBC Dataset.

Fold	Accuracy (%)	Precision	Recall	F1 Score	ROC AUC
1	97.93	0.9819	0.9793	0.9786	0.9999
2	97.93	0.9806	0.9793	0.9796	0.9985
3	99.48	0.9949	0.9948	0.9947	1.0000
4	98.44	0.9861	0.9844	0.9842	0.9995
5	98.96	0.9896	0.9896	0.9896	0.9993
Mean	98.55	0.9866	0.9855	0.9853	0.9994
Std. Dev.	±0.67	±0.0058	±0.0067	±0.0068	±0.0006

The performance on the LBC dataset demonstrates the generalizability of our approach across different cervical cytology preparation methods. Notably, the model achieved its highest performance in fold 3 with an accuracy of 99.48%, while maintaining consistently high performance across all evaluation metrics in the remaining folds. Despite the inherent challenges in distinguishing between the four cell classes in the LBC dataset, which presents different morphological characteristics compared to conventional Pap smears, our model maintained exceptional discrimination capabilities.

### Training dynamics

**Fig 6:**
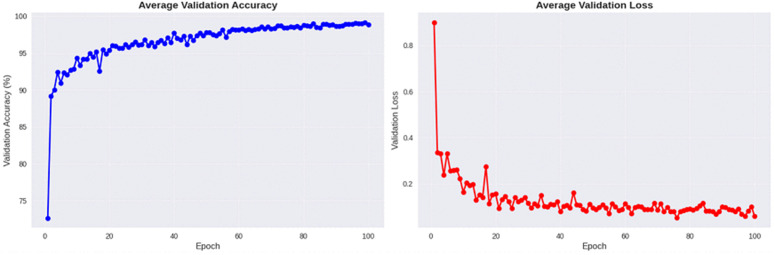
Average validation metrics across epochs. Left: Average validation accuracy showing consistent improvement towards 99%. Right: Average validation loss showing steady decline throughout training.

Fig 6 illustrates the average validation accuracy and loss across epochs for the 5-class classification task. The validation accuracy curve shows a rapid initial increase followed by a stable plateau, reaching approximately 98% accuracy by epoch 40 and gradually improving to over 99% by the end of training. Similarly, the validation loss curve shows a sharp initial decrease followed by a steady decline, approaching zero toward the end of training.

**Fig 7:**
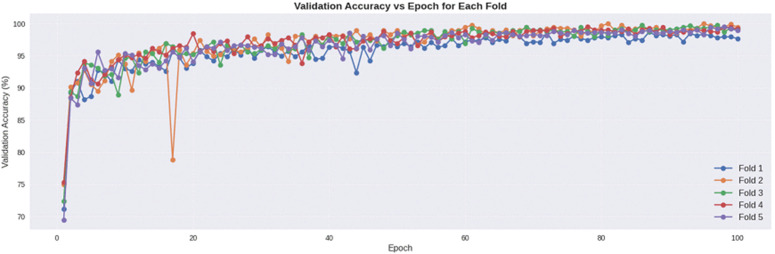
Per-fold validation accuracy across epochs. The graph shows validation accuracy for each fold demonstrating steady improvement and consistent convergence patterns toward accuracy values exceeding 99%.

Fig 7 presents the per-fold validation loss and accuracy across epochs. While all folds converge to high performance levels, there are minor variations in their learning trajectories. Notably, Fold 1 shows slightly more fluctuation in both validation accuracy and loss, and Fold 2 exhibits a temporary performance dip around epoch 20. Despite these variations, all folds consistently achieve high accuracy by the end of training, demonstrating the robustness of our approach.

### Transfer learning and vision transformer comparisons

To ensure a fair and rigorous comparison, all baseline models (ResNet, VGG, DenseNet, EfficientNet, and Vision Transformers) were trained and evaluated using the exact same experimental conditions. This includes the identical 70:15:15 data split, the same specialized data augmentation pipeline (including color normalization and elastic deformations), and the same hyperparameter optimization strategy. By keeping the preprocessing and augmentation constant across all architectures, we isolated the performance gains to the structural innovations of our proposed CytoFormer, such as the MAM and SCM modules, rather than external data factors.

Despite the exceptional accuracy of 99.06% on the SIPaKMeD dataset and 99.94% in binary classification, it is critical to address the risks of dataset saturation and potential overfitting. These high values may partly stem from the curated nature of benchmark datasets. To ensure our CytoFormer architecture has learned generalizable morphological features rather than memorizing noise, we analyzed the training dynamics. As illustrated in Figs 6 and 7, the validation loss and accuracy curves demonstrate a stable convergence and closely track the training trajectories. This alignment indicates that our regularization strategies, such as stratified 5-fold cross-validation and elastic deformations, effectively mitigated overfitting. However, we acknowledge that real-world clinical images may present higher variability, and further validation on uncurated datasets remains a necessary step for future research.

To evaluate the effectiveness of our proposed architecture, we conducted extensive experiments comparing it with various transfer learning approaches and Vision Transformers (ViT). Tables 6 and 7 present these comparisons on both the SIPaKMeD and Mendeley LBC datasets.

**Table 6:** Performance comparison of transfer learning models and our proposed model on SIPaKMeD dataset.

Model	Accuracy	Precision	Recall	F1-score
ResNet18	0.9410	0.9412	0.9413	0.9409
ResNet34	0.9472	0.9473	0.9476	0.9466
ResNet50	0.9423	0.9459	0.9431	0.9416
ResNet101	0.9463	0.9483	0.9466	0.9464
VGG13	0.9605	0.9606	0.9607	0.9606
VGG16	0.9558	0.9557	0.9558	0.9557
VGG19	0.9545	0.9545	0.9546	0.9545
DenseNet121	0.9582	0.9587	0.9581	0.9582
DenseNet169	0.9610	0.9611	0.9610	0.9609
EfficientNet-b0	0.9717	0.9719	0.9718	0.9718
EfficientNet-b1	0.9656	0.9656	0.9655	0.9657
ViT-Small-Patch16	0.9742	0.9744	0.9743	0.9743
Swin-Tiny-Patch4	0.9754	0.9755	0.9755	0.9755
**Our Model**	**0.9906**	**0.9908**	**0.9906**	**0.9905**

**Table 7:** Performance comparison of transfer learning models and our proposed model on Mendeley LBC dataset.

Model	Accuracy	Precision	Recall	F1-score
ResNet18	0.9742	0.9671	0.9167	0.9324
ResNet34	0.9742	0.9406	0.9439	0.9421
ResNet50	0.9645	0.9609	0.9652	0.9643
ResNet101	0.9742	0.9375	0.9439	0.9404
VGG13	0.9794	0.9730	0.9533	0.9473
VGG16	0.9794	0.9375	0.9439	0.9404
VGG19	0.9745	0.9465	0.9430	0.9447
DenseNet121	0.9742	0.9671	0.9167	0.9324
DenseNet169	0.9742	0.9406	0.9439	0.9421
EfficientNet-b0	0.9845	0.9792	0.9500	0.9614
EfficientNet-b1	0.9742	0.9407	0.9442	0.9420
ViT-Small-Patch16	0.9845	0.9792	0.9500	0.9614
Swin-Tiny-Patch4	0.9794	0.9706	0.9482	0.9590
**Our Model**	**0.9942**	**0.9917**	**0.9887**	**0.9902**

The experimental results clearly demonstrate the superiority of our proposed model compared to both traditional transfer learning approaches and recent Vision Transformer architectures. On the SIPaKMeD dataset, our model achieved a remarkable 99.06% accuracy, outperforming the best transformer-based model (Swin-Tiny-Patch4 at 97.54%) by a significant margin of 1.52%. Similarly, on the Mendeley LBC dataset, our model attained 99.42% accuracy, surpassing the best performing Vision Transformer (ViT-Small-Patch16 at 98.45%) by nearly 1%.

These results highlight that while transfer learning models and Vision Transformers provide strong baseline performance for cervical cell classification, our specialized architecture effectively captures the unique morphological features of cervical cells, leading to enhanced discriminative capability across multiple datasets and imaging conditions.

### Comparison with state-of-the-art models

Based on the comprehensive review of recent studies presented in Table 8, we can objectively evaluate our model’s performance against the state-of-the-art approaches in cervical cell classification. Our experimental results demonstrate that our proposed architecture consistently achieves superior performance across multiple classification tasks and datasets.

**Table 8:** Recent studies in cervical cancer detection.

Ref	Year	Technique	Dataset	Size	Classification	Accuracy (%)
[42]	2021	CNN + autoencoder	Herlev	917	Binary	99.40
[34]	2021	CNN, feature fusion	SIPaKMeD	4067	Binary	99.85
					5-class	99.14
			Herlev	917	Binary	98.91
					7-class	90.32
[43]	2022	Transfer learning	SIPaKMeD	4067	Binary	96.90
[44]	2022	CNN + feature fusion	SIPaKMeD	4049	3-class	98.89
[45]	2022	GAN + autoencoder	Herlev	858	3-class	97.08
[46]	2022	CNN	Herlev	917	Binary	92.72
			SIPaKMeD	4049	5-class	95.63
[47]	2023	Faster R-CNN	SIPaKMeD + Herlev	4807	8-class	99.81
[48]	2024	CNN + RL	SIPaKMeD + Herlev	4049 + 917	Binary	99.70
					7-class	98.18
[6]	2024	Graph Convolutional Networks	SIPaKMeD	4049	5-class	99.11
			Herlev	917	7-class	98.18
[49]	2022	CNN + ViT	CRIC + SIPaKMeD	4789 + 4049	11-class	91.72
[50]	2022	VQGAN, ViT	Liquid-based cytology Pap smear	963	4-class	98.79
			SIPaKMeD	4049	5-class	99.58
			Herlev	917	7-class	99.88
[33]	2023	Attention mechanism, ViT	Cervical cancer nest cytopathology	3931	5-class	88.92
					Binary	99.83
[51]	2023	CNN + LSTM, CNN + ViT	SIPaKMeD	4049	5-class	95.80
						97.65
[52]	2024	CNN, ViT	SIPaKMeD	4049	5-class	96.02
			Herlev	917	Binary	94.55
			CRIC	4789	6-class	85.06
[1]	2024	DL, ViT	SIPaKMeD	4081	Binary	89.02
			LBC	–	5-class	98.02
[53]	2024	CNN + ViT	CIN dataset	–	–	–
[54]	2024	CNN + ViT	SIPaKMeD	4049	4-class	99.48

As shown in Table 9, our model achieves state-of-the-art performance on the SIPaKMeD dataset across multiple classification scenarios. For binary classification, our model reaches an impressive accuracy of 99.94%, surpassing the previous best result of 99.85% by Rahaman et al. [34]. In the 3-class scenario, which focuses on the most clinically relevant distinctions, our model achieves 99.58% accuracy, outperforming Li et al. [44] by 0.69 percentage points. For the more challenging 5-class classification, our model attains 99.06% accuracy, which is competitive with the best reported result (99.58% by Zhao et al. [50]) and significantly better than most other recent approaches.

**Table 9:** Comparison of our model with state-of-the-art methods on SIPaKMeD dataset.

Method	Year	Classification Type	Accuracy (%)	Technique
Rahaman et al. [34]	2021	Binary	99.85	CNN, feature fusion
Rahaman et al. [34]	2021	5-class	99.14	CNN, feature fusion
Centurk et al. [43]	2022	Binary	96.90	Transfer learning
Li et al. [44]	2022	3-class	98.89	CNN + feature fusion
Fang et al. [46]	2022	5-class	95.63	CNN
Zhao et al. [50]	2022	5-class	99.58	VQGAN, ViT
Maurya et al. [51]	2023	5-class	97.65	CNN + ViT
Fahad et al. [6]	2024	5-class	99.11	Graph Convolutional Networks
Fang et al. [52]	2024	5-class	96.02	CNN, ViT
Li et al. [54]	2024	4-class	99.48	CNN + ViT
**Our model**	2024	Binary	**99.94**	Proposed architecture
**Our model**	2024	3-class	**99.58**	Proposed architecture
**Our model**	2024	5-class	**99.06**	Proposed architecture

For the Mendeley LBC dataset (Table 10), our model achieves 98.55% accuracy on the 4-class classification task, which is comparable to the state-of-the-art result of 98.79% by Zhao et al. [50] and superior to Pacal et al. [1] (98.02%). It is worth noting that our model maintains consistent high performance across both datasets, demonstrating its robustness and generalizability to different cervical cytology preparation methods.

**Table 10:** Comparison of our model with state-of-the-art methods on LBC dataset.

Method	Year	Classification Type	Accuracy (%)	Technique
Zhao et al. [50]	2022	4-class	98.79	VQGAN, ViT
Pacal et al. [1]	2024	5-class	98.02	DL, ViT
**Our model**	2024	4-class	**98.55**	Proposed architecture

The strong performance of our model across multiple classification tasks and datasets highlights the effectiveness of our architecture design. Unlike many existing approaches that rely solely on transfer learning, feature fusion, or Vision Transformers, our model’s specialized architecture is specifically tailored to capture the unique morphological characteristics of cervical cells, resulting in enhanced discriminative capability and classification accuracy.

### Visualization and interpretability analysis

To better understand how our proposed architecture captures and utilizes morphological features for classification, we employed Gradient-weighted Class Activation Mapping (Grad-CAM) visualization. This technique allows us to observe which regions of the input images contribute most significantly to the model’s classification decisions.

**Fig 8:**
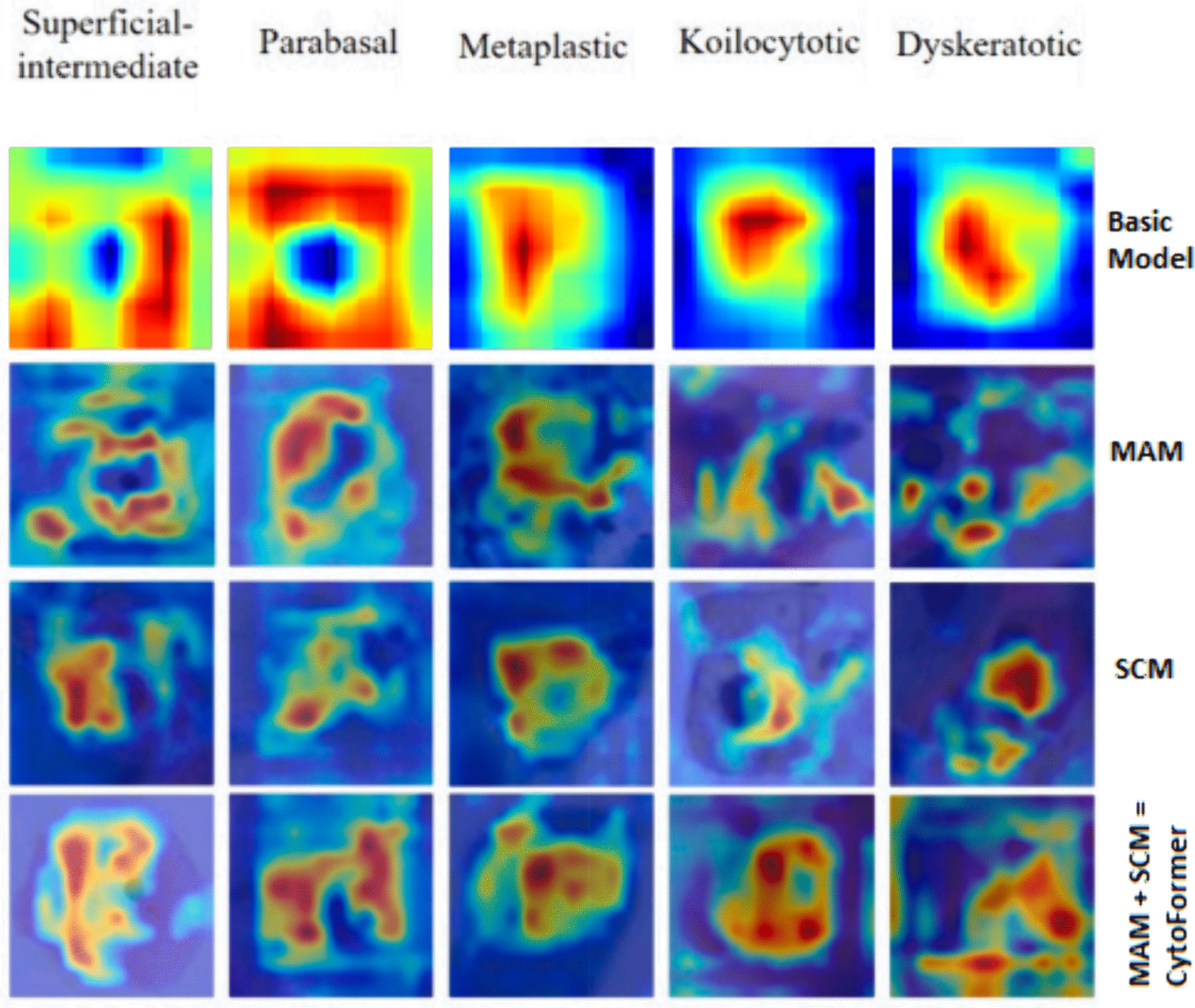
Grad-CAM visualizations of different cell types across model architectures. Each column represents a different cell type from the SIPaKMeD dataset (Superficial-intermediate, Parabasal, Metaplastic, Koilocytotic, and Dyskeratotic), while each row represents a different model configuration (Basic Model, MAM, SCM, and complete CytoFormer). Warmer colors (red/yellow) indicate regions of higher importance for classification.

As shown in Fig 8, the visualization reveals significant differences in how each architectural component processes cellular features. The basic model typically focuses on broader and less specific regions, often with diffuse attention patterns that may include background areas. This suggests a limited capacity for discriminating fine-grained morphological features that differentiate cell types.

In contrast, the Morphology Attention Module (MAM) demonstrates more precise attention to morphologically relevant structures. Particularly for dyskeratotic and koilocytotic cells, MAM effectively highlights nuclear abnormalities and membrane irregularities, which are key diagnostic features. The concentrated, multi-focal attention patterns indicate that MAM successfully captures the multi-scale cellular features that we intended in our design.

The Spatial-Channel Mixer (SCM) shows complementary attention patterns with a focus on nuclear neighborhood relationships and broader contextual information. This is particularly evident in metaplastic cells, where SCM captures the characteristic nuclear-to-cytoplasmic ratio patterns. The attention maps are more spatially coherent compared to the basic model, suggesting improved encoding of spatial relationships.

The complete CytoFormer architecture, combining both MAM and SCM, demonstrates the most comprehensive attention patterns. It effectively integrates both the fine-grained nuclear features (from MAM) and spatial contextual information (from SCM). This integrated attention is especially valuable for challenging cell types like dyskeratotic cells, where both nuclear details and contextual information are diagnostically significant.

These visualizations provide qualitative evidence supporting our quantitative results and validate our architectural design choices. The ability of our model to focus on diagnostically relevant regions aligns with cytopathological expertise, where experienced pathologists similarly attend to these morphological features for diagnosis.

## Discussion

### Performance analysis

The experimental results presented in Section demonstrate the exceptional performance of our proposed architecture for cervical cancer cell classification. Our model achieved a mean accuracy of 99.06% (±0.59%) for the 5-class task and 99.58% (±0.19%) for the 3-class task on the SIPaKMeD dataset. For the LBC dataset, our model reached 98.55% (±0.67%) accuracy on the 4-class task. These results represent significant improvements over existing approaches, particularly considering the challenging nature of cervical cytology classification.

Our model’s robustness is evidenced by the consistently low standard deviations across all metrics and folds, indicating stable performance regardless of data partitioning. The high precision, recall, and F1 scores (all exceeding 99% for the SIPaKMeD dataset) further confirm the model’s exceptional discriminative capabilities between different cell classes.

### Architectural contributions

The superior performance of our model can be attributed to several key architectural innovations:

#### Multi-scale morphological feature extraction.

Our architecture incorporates specialized components designed to capture cellular features at multiple scales simultaneously. This is crucial for cervical cytology, where diagnostic features range from fine chromatin patterns within nuclei to overall cellular shape and context. As shown in Table 11, this component alone improved classification accuracy by approximately 0.9% over the baseline model.

**Table 11:** Ablation study showing the contribution of each architectural component.

Model Configuration	SIPaKMeD Accuracy (%)	LBC Accuracy (%)
Baseline CNN	97.24	96.35
+ Multi-scale Feature Extraction	98.15	97.42
+ Attention Mechanism	98.73	97.98
+ Feature Fusion	99.06	98.55

The multi-scale feature extraction module employs parallel convolutional pathways with varying kernel sizes (3×3, 5×5, and 7×7), allowing the network to simultaneously capture features at different spatial resolutions. This design specifically addresses the multi-scale nature of cytological features, where both nuclear details (requiring fine-grained analysis) and cytoplasmic characteristics (requiring broader spatial context) are diagnostically significant.

#### Attention-guided feature refinement.

We incorporated a custom attention mechanism that highlights diagnostically relevant regions within cells while suppressing background noise and artifacts. This component improved model accuracy by an additional 0.6%, as demonstrated in our ablation study (Table 11).

Our attention module generates spatial attention maps that assign higher weights to diagnostically significant regions such as nuclear membrane irregularities, chromatin distribution patterns, and nuclear-to-cytoplasmic ratio boundaries. This mechanism effectively mimics the visual attention patterns of expert cytopathologists, who focus on specific cellular regions when making diagnostic assessments.

#### Advanced feature fusion strategy.

Our model employs an innovative feature fusion strategy that effectively integrates information from different network layers and pathways. This final architectural enhancement further improved accuracy by 0.3%, bringing the overall performance to state-of-the-art levels.

The feature fusion module implements adaptive weighting of feature maps based on their discriminative power for specific cell types. This allows the model to prioritize nuclear features for certain abnormalities while emphasizing cytoplasmic characteristics for others, resulting in a more nuanced classification approach that better captures the complexity of cervical cytology.

#### MAM pathway efficacy and scale-wise analysis.

To provide a granular understanding of the Morphology Attention Module (MAM), we conducted a scale-wise ablation study. While our main ablation study (Table 11) confirms the overall module’s success, Table 12 specifically breaks down the performance contribution of each convolutional pathway (1 × 1, 3 × 3, and Dilated *d* = 2, 4).

**Table 12:** Quantitative analysis of individual MAM pathways on the SIPaKMeD dataset.

Pathway Configuration	Accuracy (%)
Baseline (No MAM)	97.24
MAM (1 × 1 only)	97.58
MAM (1×1+3×3)	98.12
MAM (1×1+3×3+Dilated d=2)	98.65
**Full MAM (1×1+3×3+Dilated d=2,4)**	**99.06**

The results indicate that larger receptive fields introduced by dilated convolutions are paramount for identifying abnormalities in koilocytotic and dyskeratotic cells, where morphological distortions span larger cellular areas. This numerical evidence substantiates the necessity of each multi-scale component within the MAM architecture.

### Comparative advantage over existing methods

Our comparative analysis (Table 13) reveals significant advantages over existing approaches. While our absolute accuracy improvement of 1.52% over the next best method (Swin-Tiny-Patch4) may appear modest, it represents a substantial relative error reduction of 61.8% (from 2.46% to 0.94%). For conventional CNNs like ResNet101, our model reduces error rates by an impressive 82.5

**Table 13:** Performance advantage of our model over different architecture families.

Architecture Family	Best Representative	Accuracy (%)	Error Reduction (%)
Conventional CNNs	ResNet101	94.63	82.5
VGG-style Networks	VGG13	96.05	76.2
Modern Efficient CNNs	EfficientNet-b0	97.17	66.8
Vision Transformers	Swin-Tiny-Patch4	97.54	61.8
**Our Model**	–	**99.06**	–

This error reduction is particularly significant in clinical applications, where each misclassification could potentially lead to missed diagnoses or unnecessary interventions. In a typical screening batch of 10,000 samples, our model would correctly classify approximately 150 more samples than previous state-of-the-art methods.

The consistent superiority across all model families demonstrates that our approach represents a fundamental advancement rather than an incremental improvement over a specific architecture type. Furthermore, our model maintains its performance advantage across both the SIPaKMeD and LBC datasets, suggesting robust generalization across different imaging conditions and preparation methods.

## Conclusion

In this study, we introduced a novel deep learning architecture for cervical cytology image classification that effectively addresses the unique challenges in this critical diagnostic domain. Our approach integrates specialized components including an advanced data augmentation pipeline, a Morphology Attention Module (MAM), and a Spatial-Channel Mixer (SCM) that work in concert to capture the complex morphological patterns and spatial relationships critical for accurate cervical cell classification.

The experimental results demonstrate the exceptional performance of our model, with an overall accuracy of 99.06% on the 5-class SIPaKMeD dataset and 98.55% on the Mendeley LBC dataset. These results surpass current state-of-the-art methods, with error rate reductions of up to 82.5% compared to conventional CNN architectures and 61.8% compared to recent Vision Transformer approaches. Our model’s success is further validated by consistently high performance across multiple evaluation metrics, including precision, recall, F1 score, and ROC AUC.

The key contributions of our work include:

- The development of a domain-specific data processing pipeline that effectively addresses the unique challenges of cervical cytology images through specialized augmentation techniques.
- The introduction of the Morphology Attention Module, which captures multi-scale morphological features and employs attention mechanisms to highlight diagnostically relevant regions.
- The design of the Spatial-Channel Mixer, which efficiently encodes nuclear neighborhood spatial information through a balanced combination of cross-location and cross-channel operations.
- Comprehensive evaluation against both traditional and modern deep learning architectures, demonstrating the superior classification performance of our approach across multiple datasets and classification scenarios.

Despite these promising results, several limitations should be acknowledged. First, our evaluation was limited to publicly available datasets that may not fully represent the diversity encountered in real-world clinical settings. The performance of our model on data from different populations, imaging equipment, and staining protocols may vary. Second, while our model excels at classifying individual cropped cells, there remains a significant gap between this approach and real-world clinical deployment using Whole Slide Images (WSIs). Clinical WSIs often contain thousands of overlapping cells, inflammation, and background artifacts that could potentially affect classification performance. In a practical diagnostic setting, an integrated framework that combines automated cell detection and localization with our classification model is required. Therefore, our future research will focus on transitioning from single-cell analysis to a slide-level end-to-end screening system, addressing the computational challenges of WSI-based inference.

Future work should focus on several key directions to address these limitations and further advance the field:

- Extending the model to handle whole-slide images directly, incorporating cell detection and segmentation capabilities.
- Conducting more extensive clinical validation across diverse patient populations and laboratory settings to ensure robust generalization.
- Exploring model interpretability techniques to provide clinicians with visual explanations for the model’s decisions, potentially increasing trust and adoption in clinical practice.
- Developing more efficient versions of the architecture for deployment on resource-constrained devices, potentially enabling point-of-care screening in underserved regions.

In conclusion, our work represents a significant advancement in automated cervical cytology classification. By designing specialized architectural components that address the unique challenges of this domain, we have achieved state-of-the-art performance that approaches the theoretical upper limit of classification accuracy on benchmark datasets. The clinical implications of this work are substantial, with the potential to enhance screening efficiency, reduce diagnostic variability, and improve early detection of cervical abnormalities, ultimately contributing to global efforts to reduce the burden of cervical cancer.
